# Robot-Assisted, Laparoscopic, and Open Radical Cystectomy: Pre-Operative Data of 1400 Patients From The Italian Radical Cystectomy Registry

**DOI:** 10.3389/fonc.2022.895460

**Published:** 2022-05-05

**Authors:** Gian Maria Busetto, Daniele D’Agostino, Michele Colicchia, Katie Palmer, Walter Artibani, Alessandro Antonelli, Lorenzo Bianchi, Aldo Bocciardi, Eugenio Brunocilla, Marco Carini, Giuseppe Carrieri, Luigi Cormio, Ugo Giovanni Falagario, Ettore De Berardinis, Alessandro Sciarra, Costantino Leonardo, Francesco Del Giudice, Martina Maggi, Ottavio de Cobelli, Matteo Ferro, Gennaro Musi, Amelio Ercolino, Fabrizio Di Maida, Andrea Gallina, Carlo Introini, Ettore Mearini, Giovanni Cochetti, Andrea Minervini, Francesco Montorsi, Riccardo Schiavina, Sergio Serni, Claudio Simeone, Paolo Parma, Armando Serao, Mario Salvatore Mangano, Giorgio Pomara, Pasquale Ditonno, Alchiede Simonato, Daniele Romagnoli, Alessandro Crestani, Angelo Porreca

**Affiliations:** ^1^Department of Urology and Renal Transplantation, University of Foggia, Policlinico Riuniti, Foggia, Italy; ^2^Department of Urology, Villa Salus Clinic, Mestre, Italy; ^3^Department of Urology, Policlinico Abano Terme, Abano Terme, Italy; ^4^Department of Internal Medicine and Geriatrics, University Cattolica del Sacro Cuore, Rome, Italy; ^5^Department of Urology, Azienda Ospedaliera Universitaria Integrata (A.O.U.I.), Verona, Italy; ^6^Department of Urology, University of Bologna, Bologna, Italy; ^7^Department of Urology, Niguarda Hospital, Milano, Italy; ^8^Department of Urology, University of Florence, Florence, Italy; ^9^Department of Maternal-Child and Urological Sciences, Sapienza Rome University, Policlinico Umberto I, Rome, Italy; ^10^Department of Urology, European Institute of Oncology (IEO), IRCCS, Milan, Italy; ^11^Department of Urology, San Raffaele Hospital and Scientific Institute, Milan, Italy; ^12^Department of Urology, Galliera Hospital, Genoa, Italy; ^13^Department of Urology, University of Perugia, Perugia, Italy; ^14^Department of Urology, University of Brescia, Brescia, Italy; ^15^Department of Urology, Azienda Socio Sanitaria Territoriale (ASST) Mantova, Mantova, Italy; ^16^Department of Urology, Azienda Ospedaliera di Alessandria, Alessandria, Italy; ^17^Department of Urology, Ca’ Foncello Hospital, Treviso, Italy; ^18^Department of Urology, Azienda Ospedaliero-Universitaria Pisana, Pisa, Italy; ^19^Department of Emergency and Organ Transplantation, Urology, Andrology and Kidney Transplantation Unit, University of Bari, Bari, Italy; ^20^Department of Surgical, Oncological and Oral Sciences, Section of Urology, University of Palermo, Palermo, Italy; ^21^Oncological Urology, Veneto Institute of Oncology (IOV) – Istituto di Ricovero e Cura a Carattere Scientifico (IRCCS), Padua, Italy

**Keywords:** urinary bladder neoplasms, radical cystectomy, multicenter, Italy, RIC

## Abstract

**Introduction:**

The Italian Radical Cystectomy Registry (RIC) is an observational prospective study aiming to understand clinical variables and patient characteristics associated with short- and long-term outcomes among bladder cancer (BC) patients undergoing radical cystectomy (RC). Moreover, it compares the effectiveness of three RC techniques - open, robotic, and laparoscopic.

**Methods:**

From 2017 to 2020, 1400 patients were enrolled at one of the 28 centers across Italy. Patient characteristics, as well as preoperative, postoperative, and follow-up (3, 6, 12, and 24 months) clinical variables and outcomes were collected.

**Results:**

Preoperatively, it was found that patients undergoing robotic procedures were younger (p<.001) and more likely to have undergone preoperative neoadjuvant chemotherapy (p<.001) and BCG instillation (p<.001). Hypertension was the most common comorbidity among all patients (55%), and overall, patients undergoing open and laparoscopic RC had a higher Charlson Comorbidities Index (CCI) compared to robotic RC (p<.001). Finally, laparoscopic patients had a lower G-stage classification (p=.003) and open patients had a higher ASA score (p<.001).

**Conclusion:**

The present study summarizes the characteristic of patients included in the RIC. Future results will provide invaluable information about outcomes among BC patients undergoing RC. This will inform physicians about the best techniques and course of care based on patient clinical factors and characteristics.

## Introduction

Bladder cancer (BC) is the ninth most common type of cancer worldwide, and is sixth in Europe, with an age-standardized incidence of 17.7 per 100,000 and mortality rate of 5.2 per 100,000 among European men (1, 2). Tumor stage/grade is the factor most strongly predictive of outcomes even if the pathophysiology behind the aggressivity of high-risk BC has not yet been fully elucidated (3, 4). Poor clinical response of some patients can be caused by disease spread, understaging or inherent biological aggressiveness of the tumor (5, 6). Identification of new imaging strategies and prognostic biomarkers could better guide therapeutic options and identification of the molecular background of BC has improved our knowledge (tumor suppressor genes, oncogenes, cell cycle regulators, growth factors and receptors, and cell adhesion molecules) (7–10).

While recent large cohort studies have indicated that prevention of BC or prevention of advanced stages is the most effective way to reduce disease related morbidity and mortality, it remains that curative responses are necessary (11). Radical cystectomy (RC) is the only curative intervention available for advanced BC. It is performed for those patients affected by muscle-invasive bladder cancer (MIBC) or with a high-risk disease not responding to conservative treatment with trans-urethral resection followed by BCG (4). RC is a highly complex procedure, needing an experienced surgeon and centralization of care to enhance patient outcomes (12–15). Indeed, 13-67% of patients undergoing RC develop some type of complication (16–20), and while these rates of complications are high, they are also exceedingly variable.

Three techniques - open, robotic-assisted, and laparoscopic - can be used to complete RC. Robotic-assisted surgery is comparable to laparoscopic, and both seem to have better outcomes than open (21). A recent meta-analysis showed that open and robotic cystectomy have similar outcomes with regard to time to recurrence, positive margins, major com-plication rates, and quality of life; however, it seems that a robotic approach reduces the risk of blood transfusions and may reduce hospital stay (22). Even so, as of 2013, only one-quarter of RCs were being performed robotically in the US (23). Pre-surgical data, that can affect decision between techniques, are important and should be considered because can predict following complications. We speculate that a less invasive procedure can be easily performed in elderly patients, and with more comorbidities. To that end, we have designed a multicenter study in order to address these gaps in the current clinical knowledge.

The aim of the project is to investigate the pre-surgical outcomes of patients with bladder cancer who undergo radical cystectomy, comparing three different surgical techniques (robotic-assisted, laparoscopic, and open surgery).

## Material and Methods

### Study Design and Inclusion Criteria

The Italian Radical Cystectomy Registry (RIC) is an observational, prospective, multi-center, cohort study, assessing patients affected by bladder neoplasms undergoing radical cystectomy and urinary diversion *via* open, laparoscopic, or robotic-assisted technique (24). Both male and female consecutive patients are enrolled. Additionally, patients must be ≥18 years old and have histologically confirmed diagnosis of bladder cancer eligible for radical cystectomy surgery (according to EAU guidelines 2017) at enrollment. Enrollment was planned to occur from January 1^st^ 2017 to June 30th 2020, with a goal of enrolling 1000 patients, based on power calculations.

RIC is an electronic registry, and data are collected from patients at one of the 28 participating clinical centers. At each center, patient data is collected in accordance with Italian privacy law, and entered into an online database by a coordinating physician. Data collection and entering was done using the Data Collection Form, which was designed by the Scientific and Steering Committees. The Data collection form was designed using either pre-specified or open-ended responses for each question, to ensure homogeneity between centers.

Patient data are securely stored and kept anonymous using identification codes. The database is password protected. As data sharing is becoming increasingly important, the data is regularly transferred to a globally-accessible online platform. The trial has been registered retrospectively on ClinicalTrials.gov on 14/01/2020 with reference number NCT04228198. Data collection is conducted in accordance with the World Medical Association Declaration of Helsinki. This study was approved on 25/06/2020 by Ethical Committee of the University of Padova (number: 0042389). All patients provide signed informed consent.

### Participating Centers

All clinics and hospitals in Italy that currently provide care for radical cystectomy patients using all three (open, robotic, laparoscopic) techniques included in this study were invited to participate. Participation was done on a voluntary basis, without additional funding for the centers or participants. A physician at each center was assigned the role of managing patient recruitment, data collection, and the entering of the data into the registry, including maintaining data security and anonymity of the patients. Patients were enrolled at 28 centers across Italy: Urology Clinic, University of Bologna; Department of Urology, AOU Careggi, Florence; European Institute of Oncology Milan; San Raffaele Hospital, Milan; University Hospital of Verona; Department of Urology, Policlinico Abano Terme (PD); Department of Urology, Spedali Civili, Brescia; Department of Urology and Kidney Transplantation, University of Foggia, Foggia; Galliera Hospital, Genoa; ASST Niguarda Metropolitan Hospital, Milan; Policlinico Umberto I, Sapienza Rome University, Rome; Department of Clinical Urology, University of Perugia; Department of Clinical Urology, Pisa; Department of Clinical Urology, Palermo University, Palermo; Department of Clinical Urology, Alessandria Hospital, Alessandria; Department of Clinical Urology, ASST Mantova, Mantova; Department of Clinical Urology, ASL Abruzzo, Chieti; Department of Clinical Urology Ca Foncello Hospital, Treviso; Department of Clinical Urology II, Bari University, Bari; Department of Clinical Urology, Vittorio Emanuele Hospital, Catania; Department of Clinical Urology, Casa Sollievo della Sofferenza, San Giovanni Rotondo; Hospital Bassiano, Bassano del Grappa; Department of Clinical Urology, Hospital San Francesco ASL 3, Nuoro; Department of Clinical Urology, Portogruaro; Department of Clinical Urology, Biella Hospital, Biella; Department of Clinical Urology Chioggia Hospital; Ausl Modena, Modena; Department of Urology and Kidney Transplantation, Bianchi-Melacrino-Morelli Grand Metropolitan Hospital, Reggio Calabria.

### Timeline and Data Collection

**Figure 1:**
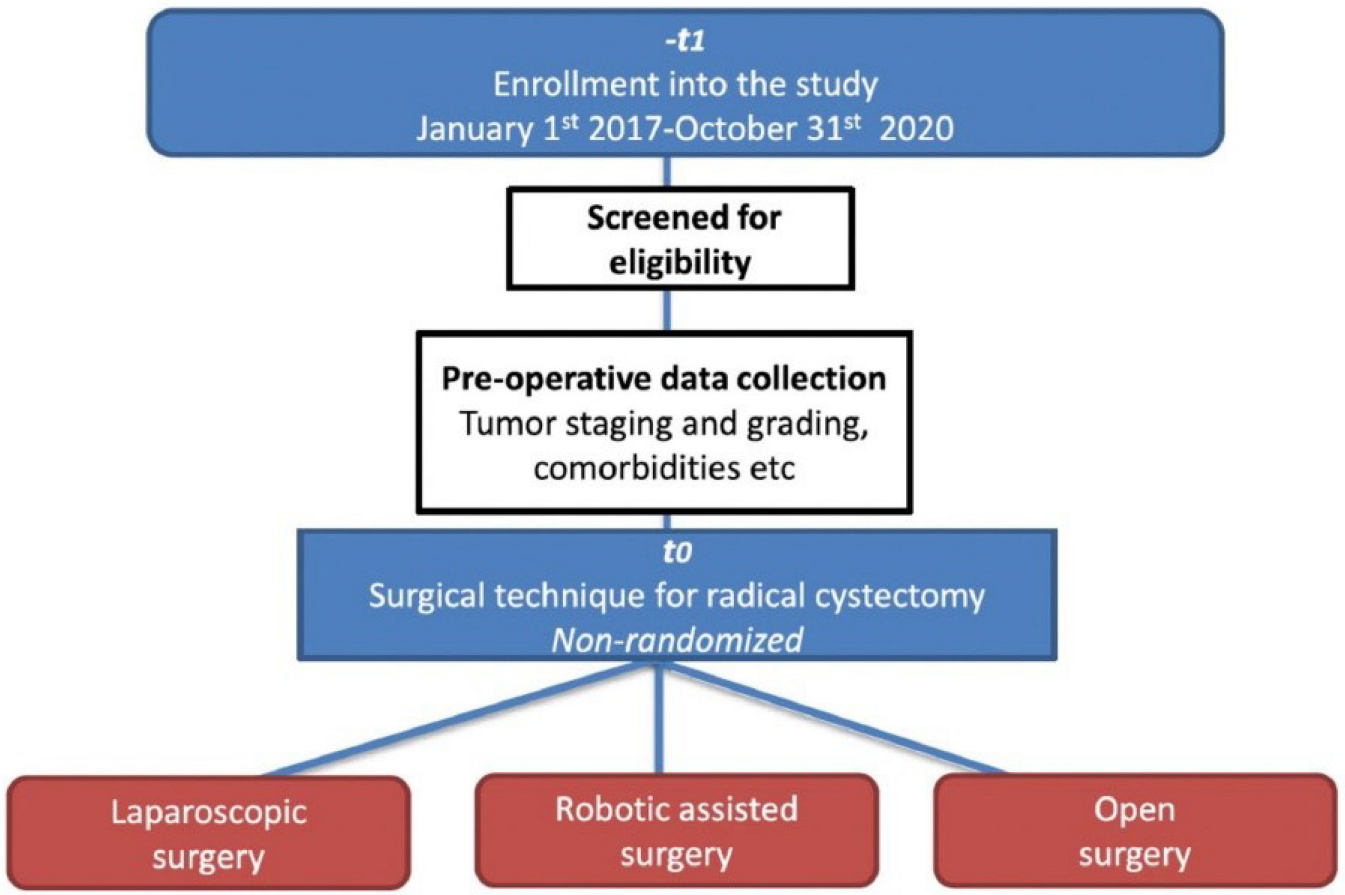
Study Phases and summary of key data.

Patient enrollment was planned for January 1^st^ 2017 to June 30^th^ 2020 (**Figure 1**). At each center, preoperative tumor staging, grading, ASA score, comorbidities, Charlson Comorbidity Index (CCI), concomitant CIS, chemotherapy use, BCG instillation, palliative cystectomy, and patient characteristics (age, sex, etc.) were collected preoperatively. Patients were operated on using laparoscopic, robotic or open surgery technique, at the discretion of the surgeon. Postoperatively, surgery time, type of urinary diversion, conversion to open surgery, presence of bleeding, nerve sparing, and lymphadenectomy were collected. Finally, follow-up care, histology (e.g., postoperative staging and grading), short-term (<30 days) (e.g., complications, readmissions, mortality), and long-term (≥24 months) (e.g., mortality, survival, sexual potency, continence) parameter were collected.

### Statistical Analysis

Data were cleaned and checked for discrepancies by a statistician before analysis and dissemination. In the case of missing data, the physician from the respective center was contacted and asked to review medical records and data sheets for the missing information, to minimize missingness. In case was impossible to find the value of a missing data the patient was excluded from analysis of that specific parameter. Logistic regression was used to analyze data, while chi-square and t-tests to compare categorical and continuous variables, respectively, between surgical technique groups. Statistical analyses were performed using Stata-SE 15 (StataCorp LP, College Station, TX, USA). All tests were 2-sided with a significance level set at p<0.05.

## Results

**Figure 2:**
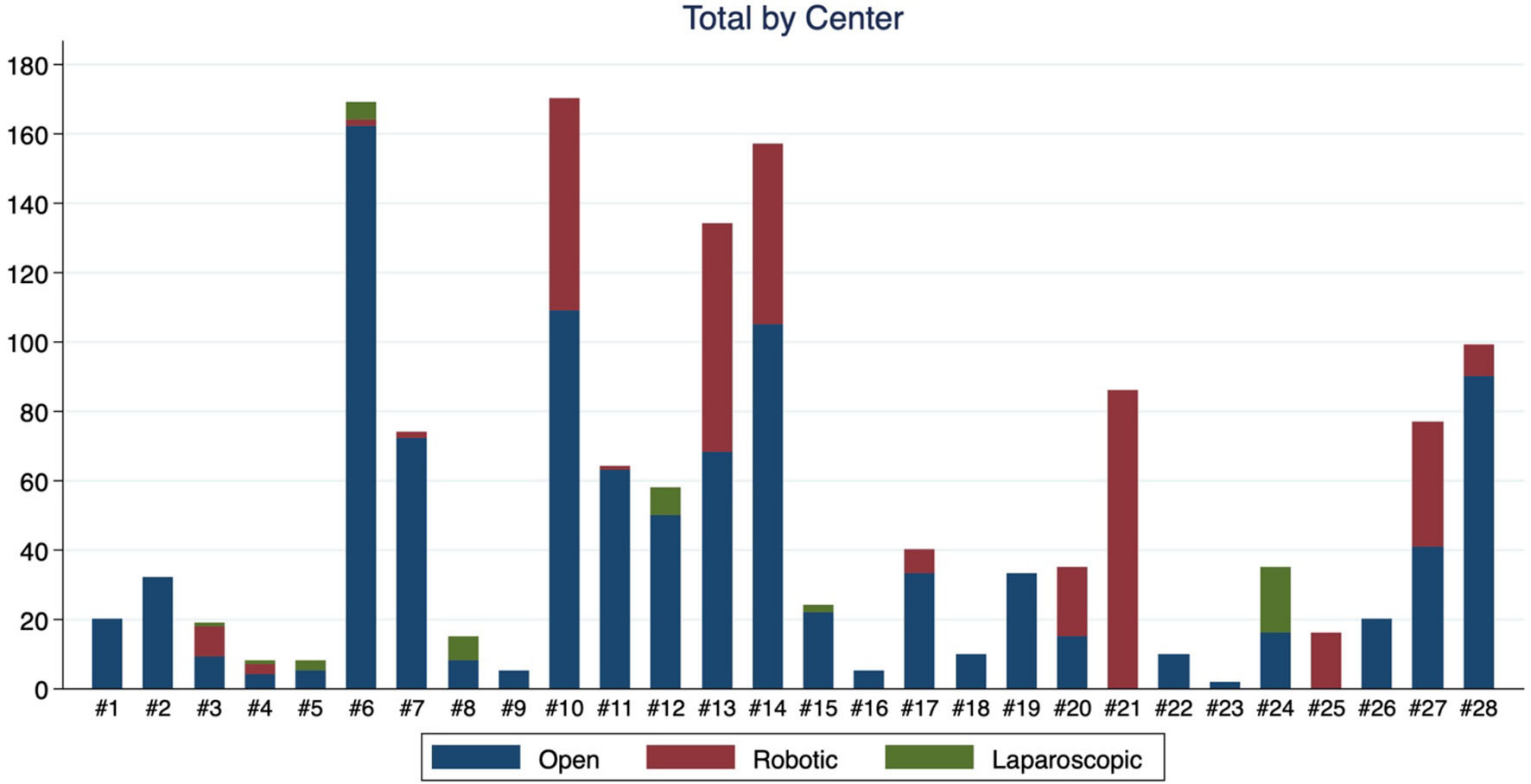
Case volume of RC in the Italian Radical Cystectomy trial in 28 medical centers.

An enrollment of 1000 patients was planned, but it was closed on February 2020 because, surprisingly, included patients exceeded original estimates. A total of 1400 patients undergoing radical cystectomy and urinary diversion *via* open, laparoscopic, or robotic-assisted technique were included. Patient enrollment, by center and technique, is detailed in **Figure 2** and **Table 1**. Patients had a mean age of 69.9 years (SD 10.0) and 18.7% were female. Hypertension (55%), followed by anticoagulant use (35.1%) and other pathologies (35.4%) were the most common comorbidities. Preoperatively, nearly all (92.6%) were classified as grade 3 and two-thirds (65.7%) as pT2. Preoperative patient characteristics of the overall population are presented in **Table 2**.

**Table 1:** General characteristics of the 1400 patients in the Italian Radical Cystectomy trial: number of surgical techniques used for radical cystectomy (open, robotic, laparoscopic) in 28 medical centers.

	Open		Robotic		Laparoscopic		Total	
	n	%	n	%	n	%	n	*%*
**All hospitals**	999	71.36	354	25.29	47	3.36	1400	100.00
**Center # 1**	21	100.00	0	0.00	0	0.00	21	1.50
**Center # 2**	32	100.00	0	0.00	0	0.00	32	2.29
**Center # 3**	9	47.37	9	47.37	1	5.26	19	1.36
**Center # 4**	4	50.00	3	37.50	1	12.50	8	0.57
**Center # 5**	5	62.50	0	0.00	3	37.50	8	0.57
**Center # 6**	162	95.86	2	1.18	5	2.96	169	12.07
**Center # 7**	73	97.33	2	2.67	0	0.00	75	5.36
**Center # 8**	9	56.25	0	0.00	7	43.75	16	1.14
**Center # 9**	5	100.00	0	0.00	0	0.00	5	0.36
**Center # 10**	107	63.69	61	36.31	0	0.00	168	12.00
**Center # 11**	63	98.44	1	1.56	0	0.00	64	4.57
**Center # 12**	50	86.21	0	0.00	8	13.79	58	4.14
**Center # 13**	68	50.75	66	49.25	0	0.00	134	9.57
**Center # 14**	105	66.88	52	33.12	0	0.00	157	11.21
**Center # 15**	22	91.67	0	0.00	2	8.33	24	1.71
**Center # 16**	5	100.00	0	0.00	0	0.00	5	0.36
**Center # 17**	34	82.93	7	17.07	0	0.00	41	2.93
**Center # 18**	10	100.00	0	0.00	0	0.00	10	0.71
**Center # 19**	33	100.00	0	0.00	0	0.00	33	2.36
**Center # 20**	15	42.86	20	57.14	0	0.00	35	2.50
**Center # 21**	0	0.00	86	100.00	0	0.00	86	6.14
**Center # 22**	10	100.00	0	0.00	0	0.00	10	0.71
**Center # 23**	3	100.00	0	0.00	0	0.00	3	0.21
**Center # 24**	17	45.95	0	0.00	20	54.05	37	2.64
**Center # 25**	0	0.00	16	100.00	0	0.00	16	1.14
**Center # 26**	20	100.00	0	0.00	0	0.00	20	1.43
**Center # 27**	27	57.45	20	42.55	0	0.00	47	3.36
**Center # 28**	90	90.91	9	9.09	0	0.00	99	7.07

**Table 2:** Pre-operative characteristics of all patients (n=1400).

	Mean	*SD*
Age	69.9	10.0
	**n**	**%**
Female	262	18.7
Charlson Comorbidity Index (CCI)	3.7	2.5
Diabetes	243	17.4
Hypertension	770	55.0
Cardiopathy	344	24.6
COPD	202	14.4
TIA	71	5.1
Anticoagulants	492	35.1
Other pathologies	495	35.4
ASA score (missing n=86)		
1	106	8.1
2	575	43.8
3	569	43.3
4 or 5	64	4.9
Preoperative T-stage (missing n=42)		
Ta	50	3.7
T1	263	19.4
T2	892	65.7
T3	65	4.8
T4a	35	2.6
T4b	10	0.7
TIS	43	3.2
Preoperative G-stage (missing n=58)		
G0 (undetermined)	14	1.0
G1	19	1.4
G2	66	4.9
G3	1243	92.6
Preoperative Concomitant CIS (missing n=42)	216	15.9
Preoperative neoadjuvant chemotherapy (missing n=26)	158	11.5
Preoperative BCG instillation (missing n=28)	281	20.5
Preoperative palliative cystectomy (missing n=38)	129	9.5

In comparison of patients by surgical technique, significant differences were found. Roughly three-quarters of patients (n=999) underwent open surgery, while 25.3% (n=354) underwent robotic surgery, and the remaining 3.4% (n=47) underwent laparoscopic surgery (**Table 3**). Age differed significantly by group (p<.001), with those undergoing robotic surgery being the youngest. Patients also varied significantly on all comorbidities (and Charlson Comorbidity Index) except TIA. Additionally, there were differences in preoperative ASA score (p<.001), with a trend for open surgery patients to have a higher score. Similarly, they differed on preoperative G-stage classification (p=.003), with more laparoscopic patients being classified as G2, versus G3, which was more common among open and robotic patients. Moreover, patients who underwent robotic surgery had higher rates of preoperative neoadjuvant chemotherapy (p<.001) and BCG instillation (p<0.001), while open surgery patients had higher rates of palliative cystectomy (p<.001). Patients did not differ between groups on preoperative T-stage (p=.128) or concomitant CIS (p=.136).

**Table 3:** Pre-operative characteristics of patients according to surgical technique.

	Open (n=999)		Robotic (n=354)		Laparoscopic (n=47)		P value
	Mean/n	SD/%	Mean/n	SD/%	Mean/n	SD/%	
Age	71.2	9.5	65.9	10.1	71.8	10.0	<.001
	**n**	**%**	**n**	**%**	**n**	**%**	
Female	204	20.4	51	14.4	7	14.9	.039
Charlson Comorbidity Index (CCI)	4.04	2.6	2.88	2.14	4.15	2.4	<0.001
Diabetes	184	18.4	46	13	13	27.7	.011
Hypertension	569	57	172	48.6	29	61.7	.016
Cardiopathy	272	27.2	55	15.5	17	36.2	<.001
COPD	162	16.2	32	9	8	17	.004
TIA	57	5.7	12	3.4	2	4.3	.225
Anticoagulants	372	37.2	97	27.4	23	48.9	.001
Other pathologies	384	38.4	87	24.6	24	51.1	<.001
ASA score (missing n=86)							<.001
1	43	4.5	61	20	2	4.3	
2	391	40.6	160	52.5	24	51.1	
3	471	49	81	26.6	19	36.2	
4 or 5	57	5.9	3	1	4	8.5	
Preoperative T-stage (missing n=42)							.128
Ta	35	3.6	14	4.1	1	2.2	
T1	194	19.9	58	17.1	11	23.9	
T2	627	64.4	237	69.9	28	60.9	
T3	47	4.8	14	4.1	4	8.7	
T4a	32	3.3	2	0.6	1	2.2	
T4b	10	1	0	0	0	0	
TIS	28	2.9	14	4.1	1	2.2	
Preoperative G-stage (missing n=58)							.003
G0 (undetermined)	9	0.9	5	1.5	0	0	
G1	17	1.8	2	0.6	0	0	
G2	43	4.5	15	4.5	8	17.4	
G3	895	92.8	310	93.4	38	82.6	
Preoperative Concomitant CIS (missing n=42)	150	15.3	62	18.6	4	8.5	.136
Preoperative neoadjuvant chemotherapy (missing n=26)	88	8.9	70	20.6	0	0	<.001
Preoperative BCG instillation (missing n=28)	182	18.4	95	28.3	4	8.5	<.001
Palliative cystectomy (missing n=38)	109	11.1	12	3.6	8	17	<.001

The multivariable analysis predicting minimally invasive radical cystectomy (Open vs Robotic+ Laparoscopic) has been performed and reported in **Table 4**. Age and CCI were independently associated with minimally invasive surgery.

**Table 4:** Multivariable analysis predicting minimally invasive radical cystectomy (Open vs Robotic+ Laparoscopic).

Covariate	Odds Ratio	95% Conf. Interval	P>|z|
**Age**	0.97	0.95,0.98	<0.001
**Gender**			
Female	Ref.		
Male	1.92	1.31,2.82	0.001
**Charlson Comorbidity Index (CCI)**	0.94	0.88,1.01	0.095
**ASA score**			
1	Ref.		
2	0.42	0.26,0.66	<0.001
3	0.24	0.14,0.40	<0.001
4 or 5	0.18	0.07,0.48	0.001
**Preoperative T-stage**			
<T2	Ref.		
T3	0.81	0.37,1.76	0.587
T4a	1.39	0.66,2.92	0.385
T4b	1.48	0.58,3.73	0.409
**Neoadjuvant chemotherapy**			
No	Ref.		
Yes	1.02	0.33,2.65	0.876
**Preoperative BCG instillation**			
No	Ref.		
Yes	1.00	0.34,2.95	0.998
**Palliative cystectomy**			
No	Ref.		
Yes	1.64	1.10,2.44	0.016

## Discussion

This first analysis from the Italian Radical Cystectomy Registry (RIC) reported preoperative results of advanced BC patients, comparing three RC techniques - open, robotic-assisted, and laparoscopic. This study was designed to assess, in a real-world context, factors associated with disease-related morbidity and mortality and effectiveness of the separate RC techniques. We found that among BC patients undergoing RC, those undergoing robotic procedures were younger and more likely to have undergone preoperative neoadjuvant chemotherapy and BCG instillation. Additionally, patients undergoing open and laparoscopic RC had a higher Charlson Comorbidities Index compared to patient undergoing robotic RC (p<.001). There were also significant differences in terms of grading and staging between patient groups, including laparoscopic patients having a lower G-stage classification and open patients having a higher ASA score. This suggests that laparoscopic patients, at least compared to open patients, have less advanced disease at the time of surgery.

In our study, BC patients undergoing robotic RC were younger. Conversely, Rai et al., in a Cochrane Review comparing robotic versus open radical cystectomy, showed no differences in patients age between robotic and open group (22).

These preliminary findings from this large prospective cohort study suggest that differences observed in disease-related morbidity and mortality between RC techniques may be related to preoperative patient clinical factors and characteristics. Although past studies have suggested that outcomes are similar among patient who undergo robotic and laparoscopic RC, some of these differences may be due to preoperative differences be-tween groups (21, 22). Currently, one study is attempting to disentangle what may be due to inherent differences versus technique in a randomized comparative effectiveness trial (24). However, it must be noted that surgeons must evaluate the safest and most effective technique for the patient based on a myriad of real-world factors. Although trending evidence suggests that robotic or laparoscopic surgery is preferable to open surgery, only 25% of BC patients undergo robotic surgery in the US (23), which is consistent with our findings. Therefore, understanding why most surgeries are still being done using an open technique and how this may affect patients is critical to improving outcomes.

This study has several strengths, including a large representative sample, a longitudinal design, and a real-world contextual setting. However, we must also consider that there are surgeon biases that may affect technique choice, which cannot be controlled. In fact, the decision between one technique and the others was completely under surgeon discretion. The difference in procedure and protocols among different institutions (which however is common in every multicenter study) is another potential selection bias. Additionally, although this sample is representative of southern Europe, the highest BC incidence and mortality rates are in high human development index countries (1), so findings from this study and future studies in the RIC may not be generalizable to these populations. Finally, because laparoscopic procedures are still uncommon, there were few patients (3.4%) in our cohort who underwent this type of intervention. We even believe that laparoscopic RC is the most complex technique, and probably for this reason the less performed. Therefore, power may have been and may be limited in analyzing results in this group. However, this study was designed to have sufficient power and, indeed, exceeded enrollment targets.

## Conclusion

This study presented preliminary, preoperative results from RIC. RIC is even a prospective longitudinal study that will follow patients postoperatively and over the follow-up care period. As the study progresses, we will be able to investigate clinical factors and patient characteristics associated with both short- and long-term outcomes, as well as differences in outcomes based on technique used. This study will provide invaluable information that will help to inform physicians about the best care plans for advanced BC patients to reduce disease-related morbidity and mortality.

## Data Availability Statement

The raw data supporting the conclusions of this article will be made available by the authors, without undue reservation.

## Ethics Statement

The study involving human participants was reviewed and approved by Ethical Committee of University of Padua (number: 0042389). The patients/participants provided their written informed consent to participate in this study.

## Author Contributions

AA, AB, RS, LC, and AM are on the Scientific Committee for the current project. WA and AP are on the steering committee for the current project. GMB, DD’A, MicC, KP, WA, AA, LB, AB, EB, MarC, GiuC, LC, UGF, EDB, AleS, CL, FDG, MM, OdC, MF, GM, AE, FDM, AG, CI, EM, GioC, AM, FM, RS, SS, CS, PP, ArmS, MSM, GP, PD, AlcS, DR, AC, AP provided patient data for the multicenter trial described in the article. All authors provided input into the planning of the protocol including the definition of inclusion criteria, variables and data collection. GMB, MicC, FDG, UGF and KP wrote the manuscript draft. All authors revised the draft.

## Funding

The Italian Radical Cystectomy Registry is an observational, non-profit study. AB Medica provided an unconditional grant for the maintenance of the database. AB Medica has no involvement in the acquisition, control, or management of the data. The registry and all data are exclusively owned by the Steering Committee.

## Conflict of Interest

The authors declare that the research was conducted in the absence of any commercial or financial relationships that could be construed as a potential conflict of interest.

## Publisher’s Note

All claims expressed in this article are solely those of the authors and do not necessarily represent those of their affiliated organizations, or those of the publisher, the editors and the reviewers. Any product that may be evaluated in this article, or claim that may be made by its manufacturer, is not guaranteed or endorsed by the publisher.
